# The Plastic Surgeon’s Role in the COVID-19 Crisis: Regarding Domestic Violence

**DOI:** 10.7759/cureus.12650

**Published:** 2021-01-12

**Authors:** Olivia Abbate Ford, Bharti Khurana, Indranil Sinha, Matthew J Carty, Dennis Orgill

**Affiliations:** 1 Plastic Surgery, Harvard University, Boston, USA; 2 Radiology, Brigham and Women’s Hospital, Boston, USA; 3 Division of Plastic and Reconstructive Surgery, Brigham and Women's Hospital, Boston, USA; 4 Division of Plastic and Reconstructive Surgery, Brigham and Women’s Hospital, Boston, USA

**Keywords:** intimate partner violence, domestic violence, craniofacial, trauma, hand

## Abstract

Pandemics are associated with increased rates of intimate partner violence (IPV). IPV-related physical abuse is most commonly inflicted through craniofacial assault and upper extremity injury. Plastic surgeons are frequently consulted for recommendations in the management of head-and-neck and hand trauma, thereby are uniquely positioned to encounter patients who have experienced IPV. However, IPV training is not routinely offered in surgical education. We provide a review of the increasing prevalence of IPV during the COVID-19 pandemic and its pertinence to plastic surgery consultation in the emergency room. This article aims to increase providers' confidence in recognizing IPV-suspicious injuries and propose an educational, interactive tool for discussing IPV with patients.

## Introduction and background

There has been a reported increase in the incidence of domestic violence on a global scale since the start of the COVID-19 pandemic. Intimate partner violence (IPV), a subtype of domestic violence involving physical or sexual violence from a current or former partner, is estimated to affect one in five women and one in seven men in a non-pandemic lifetime [1,2]. IPV survivors often incur chronic physical and mental health problems including physical disabilities and PTSD [1,2]. Physical signs of IPV are most evident through head-and-neck trauma and upper extremity injury. Plastic surgeons are frequently consulted for recommendations in the management of craniofacial and hand trauma, thereby are uniquely positioned to encounter and identify victims of IPV. In this article, we review the prevalence of IPV and its increasing manifestations during the COVID-19 pandemic, present two cases of IPV requiring plastic surgical treatment at our own institution, and highlight the role plastic surgeons may play in diagnosing IPV during routine trauma care. We also propose a simple interaction model to help plastic surgeons approach cases of IPV.

## Review

Methods & materials

A comprehensive literature search of PubMed/MEDLINE and Google Scholar databases was conducted from May to August 2020. Databases were searched in varying combinations of keywords “domestic violence” “intimate partner violence” “COVID-19” “craniofacial trauma” “upper extremity trauma”. Forty publications were collected manually and after meticulous review, 27 contained information and data relevant for inclusion in this article.

IPV and COVID-19

Pandemics are routinely and historically associated with increases in the reports of DV [3,4], and COVID-19 has shown early signs of similar patterns [4,5]. Compared to February 2019, February 2020 has shown three times the number of domestic violence reports in the Hubei province in China [5,6]. Emergency hotlines in Spain report a nearly 20% increase in domestic violence (DV) calls, France reports a 30% increase, while Italy’s police department has been helping victims requisition hotel rooms [5,6]. In the United States, data is evolving. Early reports of DV increased by 5% in Chicago, Kansas City, Los Angeles, Memphis, and New Orleans [6,7]. New York City and Boston have reported a decline in DV calls to city police, despite an increase in DV reports statewide and an increase in calls to shelters [8,9]. Local governments express concern that this dip is reflective of barriers reaching law enforcement, rather than a true decline in cases [8,9]. 

Pandemics prove a uniquely difficult circumstance to victims of IPV. Factors that increase a person/relationship risk for IPV include lower socioeconomic status, lower education level, substance abuse, financial dependency, and job stress [10-12], all of which are magnified under an emergency crisis setting. The Center for Global Development reports discrete ways in which a pandemic can facilitate IPV; most notably economic insecurity and poverty-related stress, social isolation, and reduced availability of health services and screening [13]. Past pandemics have shown how these temporary changes to social and economic structures, while often necessary to contain the spread of disease, can create dysfunctional home environments for those at risk [14]. Victims are spending more time than ever at home with their partners, with decreased connections to social networks and support systems, less interactions with family, friends, and communities, increased financial stressors, and heightened fear of illness [13-14]. IPV flourishes in these settings because they create an unescapable situation - relationships, churches, hospitals that once served as havens are now unsafe or unavailable, and we cannot predict with certainty when they will be accessible once again. The Deputy Director of the United Nations Women, Anita Bhatia, states, “while we absolutely support the need to follow these measures of social distancing and isolation, we also recognize that it provides an opportunity for abused to unleash more violence” [6]. The World Health Organization has declared that health care systems in the United States must prepare for an increase in DV in our communities [14].

IPV patient cases

IPV assaults can be classified as “target” or “defensive”; target injuries are sustained from an offensive attack and defensive injuries are sustained from an attempt to block an attack. The face is the most common target area while the upper extremity is the most common defensive area [12]. Head-and-neck trauma is most common overall, followed by upper trunk/extremity injury [15-18], with the majority of patients presenting with contusions/abrasions, lacerations, sprains, organ injury, and fracture, respectively. More than 80% of non-sexual IPV assaults in the ED result in facial injury [18]. Patients presenting to the emergency department with head and neck trauma are 11.8 times more likely to be victims of domestic violence than those seeking treatment for other injuries [18]. Compared to trauma in women related to falls or motor-vehicle accidents, IPV trauma is more likely to present as an isolated injury, on the left side, with a predilection for younger age [18, 19]. Punching is the most common mechanism of injury and it is theorized that because most of the population is right-hand dominant most fists are delivered to the left face [18-20]. Household items such as bottles and pipes are also not infrequently used [20,21]. The most common fractures include nasal bone, zygoma, orbit, and then mandible; secondary to facial fractures, fractures of the upper extremity and trunk are most common [18].

**Figure 1:**
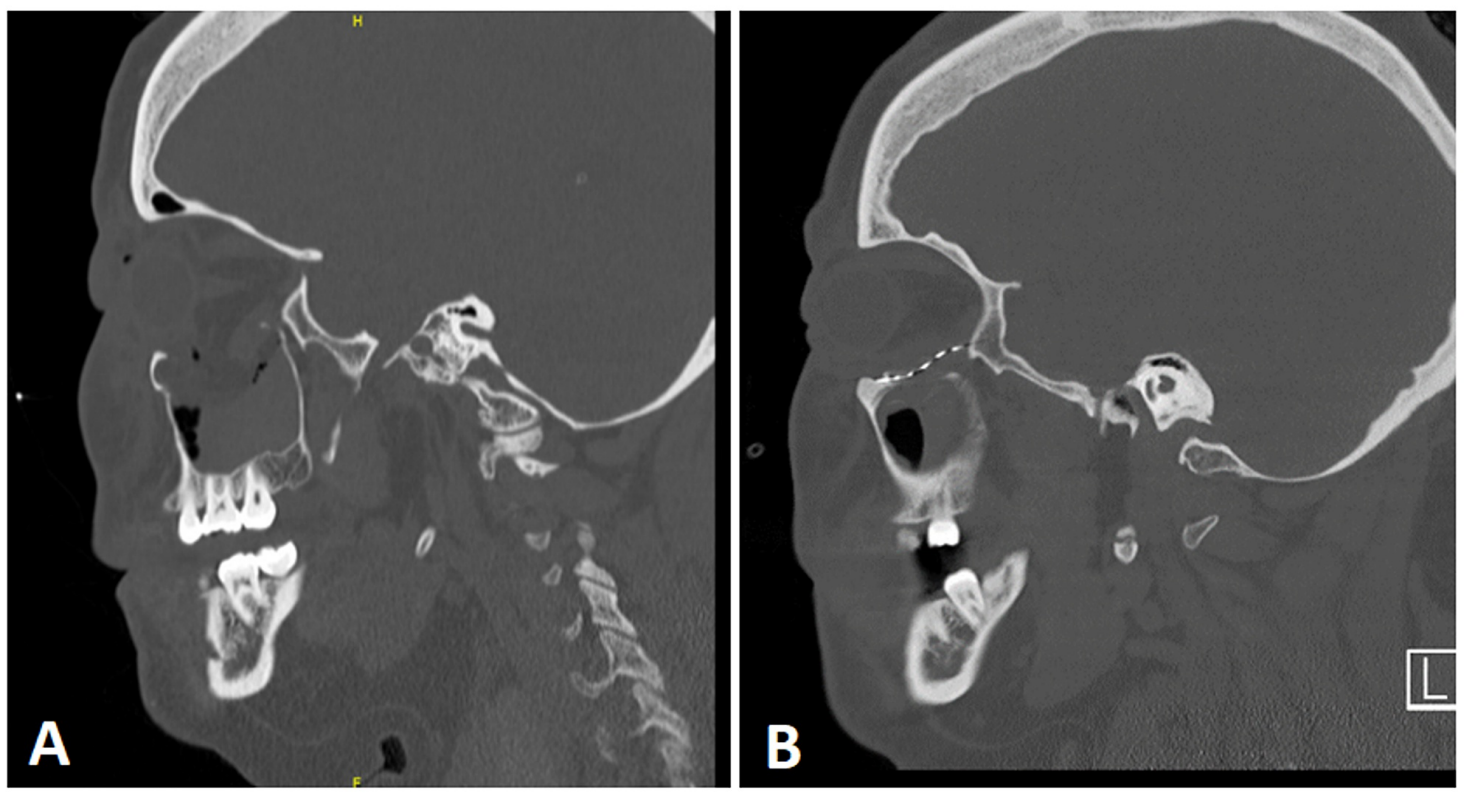
Young female with orbital floor fracture from IPV. IPV: intimate partner violence

We present below two cases of IPV recognized by plastic surgery providers in our institution's ED during the COVID-19 quarantine. The first patient is a young female who was assaulted by her husband during the COVID-19 pandemic. The couple was arguing at home when he began blunting attacking her in the face until she became unconscious. On arrival at the ED, she had pain in the right eye and difficulty opening the lid. On examination, she had edema and ecchymosis of the periorbita with subconjunctival hematoma. Her visual acuity was normal, extraocular muscles were intact without evidence of entrapment. CT scan (Figure 1A) demonstrated a comminuted displaced fracture of the inferomedial orbital wall resulting in a 2x2cm orbital floor defect. The patient disclosed IPV voluntarily and underwent debriefing with social work and the local police department, which is our institution's standard protocol. Upon investigation, it was discovered that this was not the patient’s first incidence of IPV requiring treatment in the ED. The patient underwent ORIF of the orbital floor with excellent reconstitution of her facial skeleton (Figure 1B).

**Figure 2:**
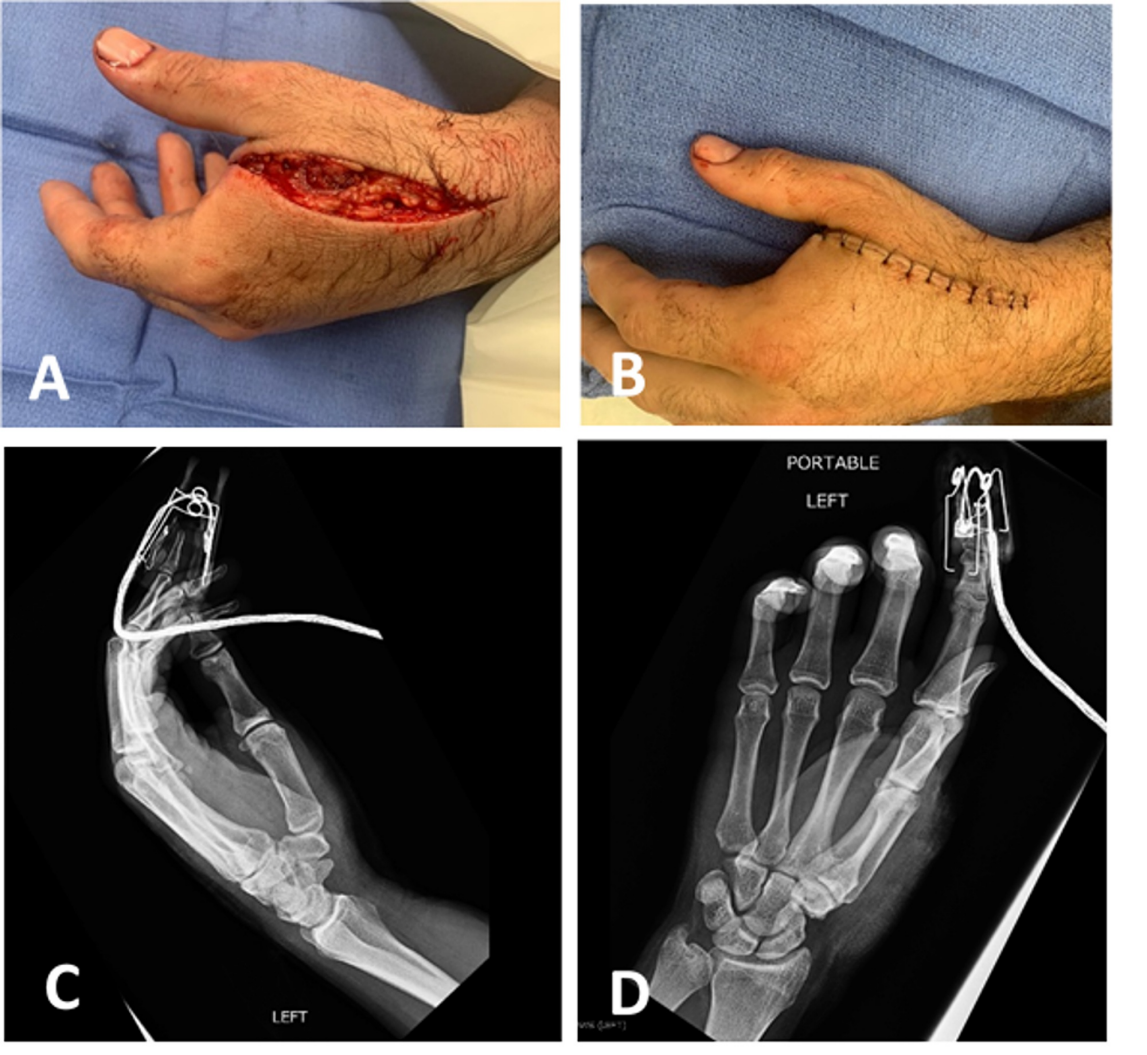
Young male with a hand injury from IPV. IPV: intimate partner violence

The second patient is a young, right-hand dominant male mechanic who sustained a stab injury to the left hand after a domestic dispute during the COVID-19 quarantine. The patient voluntarily disclosed that he was arguing with his girlfriend when she began stabbing him with a kitchen knife. The patient placed his hands up to protect himself which resulted in a 10cm laceration through the dorsal first web space of the left hand (Figure 2A). On examination, the first dorsal interosseous muscle and adductor pollicis were transected. He had minimally decreased sensation over the radial aspect of the index finger. X-rays were negative for fracture (Figures 2C, 2D). He was given a tetanus booster, and wounds were irrigated and repaired at the bedside in the ED (Figure 2B). He was placed in a thumb spica splint and sent to the observation unit for social work evaluation. He was able to secure safe housing with a friend.

As the only specialty with specialized training in both of these systems - craniofacial and upper extremity - plastic surgeons are uniquely positioned to encounter IPV. However, IPV is not part of routine surgical training. Surgeons have cited “lack of knowledge”, “lack of training”, “time constraints”, “personal discomfort”, and “fear of offending or insulting patients” as reasons for not inquiring about IPV [16,22]. Only 14% of patients presenting with injuries from IPV are asked about partner violence by health professionals [12]. Previous studies have shown that only a minority of people who experience IPV are asked about partner violence by their generalists or by the ED staff [23,24]. In the United States, routine IPV screening is now recommended in the primary care setting as it has been shown to decrease the risks of subsequent violence [25]. Plastic surgeons may be the second-line provider to identify IPV during trauma consultation in patients that were not asked about IPV by their generalists or ED [23,24].

Recommendations

Physicians who receive IPV training are more likely to demonstrate positive attitudes about their ability to diagnose and assist patients with suspicious injuries [16]. At our own institution, we propose the following interaction model for how plastic surgeons can approach cases of IPV (Table 1).

**Table 1:** Interaction model for plastic surgeons and patients at risk for IPV. Note: Original model created by authors, with language and recommendations adapted from Refs. [14,25]. IPV: intimate partner violence

Phase of Approach	Details of Interaction
Identify	If you see a patient with isolated face or hand trauma, consider IPV in your differential
Inquire	Create a safe space for discussion which includes an interview and exam alone, without the partner present Use routine practice qualifying statements; “It is our practice to routinely ask all patients...” or “I have patients with similar injuries who were hit or attacked by their partners …” Avoid phrases such as “victim” or “battered”, instead try using “partner in relationship” “patient” or “recipient” If a patient denies IPV, respect their wishes
Provide Support	Thank the patient for their honesty, reassure them of your confidentiality Ask the patient if they give permission to be seen by social work, "We routinely ask our social work team to help us figure out the best way to help you, we recommend that they be part of your care team today" If patient is agreeable, consult the social work team, if patient is not agreeable, respect their wishes If there is no social work team, discuss with the emergency department
Provide Resources	The National Domestic Violence Hotline is reachable 24/7 at 1-800-799-SAFE (7233) or online at www.thehotline.org Be sure to document professional medical opinion with objective language and data. Should the patient eventually pursue legal action the electronic medical record can be utilized in court of law

At our institution, safety and housing details are looked into by the social work team, however, we recognize that this resource is not available everywhere. Therefore, it may be important to discuss with the ED to become familiar with shelters and safe spaces that are open locally as well as resources online. The WHO website offers several picturesque educational handouts for patients seeking guidance, as well as for instructions for physicians on reporting cases [26]. The National Domestic Violence Hotline advocates are available 24/7 at 1-800-799-SAFE (7233) [27]. 

There are also several learning platforms designed specifically for training health care professionals in IPV that can be done online. EDUCATE is a program created to provide orthopedic surgeons with knowledge and skills to assist women who are victims of IPV [16]. The WHO also offers ample education material in the form of handouts online; available in supplemental material [26]. In the future, our hope is for all surgical subspecialties to include some form of IPV training for trainees and faculty.

## Conclusions

As the COVID-19 pandemic continues to evolve, we are asked to adapt, change, and reflect on our own practices to meet the demands of a changing healthcare delivery system. Domestic violence is a common, devastating physical and psychological insult on society that is increasing in the current climate. Plastic surgeons are frontline responders to victims of craniofacial and extremity trauma, which are common IPV-related injuries. We hope that this article has provided substantive evidence that IPV is a relevant, prevalent, and predictable condition that Plastic Surgeons encounter and have the potential to impact during the current COVID-19 pandemic and thereafter.
